# An Examination of the Motives for Attributing and Interpreting Deception in People with Amnestic Mild Cognitive Impairment

**DOI:** 10.3390/jintelligence12020012

**Published:** 2024-01-24

**Authors:** Maria Tilkeridou, Despina Moraitou, Vasileios Papaliagkas, Nikoleta Frantzi, Evdokia Emmanouilidou, Magdalini Tsolaki

**Affiliations:** 1 Neurosciences and Neurodegenerative Diseases, Postgraduate Course, Medical School, Faculty of Health Sciences, Aristotle University, 54124 Thessaloniki, Greece; tsolakim1@gmail.com; 2 Greek Association of Alzheimer’s Disease and Related Disorders (GAADRD), 54643 Thessaloniki, Greece; 3 Laboratory of Psychology, Department of Experimental and Cognitive Psychology, School of Psychology, Faculty of Philosophy, Aristotle University, 54124 Thessaloniki, Greece; nkfrantzi@gmail.com (N.F.);; 4 Laboratory of Neurodegenerative Diseases, Center for Interdisciplinary Research and Innovation (CIRI-AUTH), Balkan Center, Aristotle University, 10th km Thessaloniki-Thermi, 54124 Thessaloniki, Greece; 5 Department of Biomedical Sciences, School of Health Sciences, International Hellenic University, 57400 Thessaloniki, Greece; vpapaliagkas@gmail.com

**Keywords:** deception, dishonesty, executive functions, theory of mind (ToM)

## Abstract

The aim of the present study was to examine how a person with amnestic mild cognitive impairment perceives the phenomenon of deception. Amnestic mild cognitive impairment (aMCI) usually represents the prodromal phase of Alzheimer’s disease (AD), with patients showing memory impairment but with normal activities of daily living. It was expected that aMCI patients would face difficulties in the attribution and interpretation of deceptive behavior due to deficits regarding their diagnosis. The main sample of the study consisted of 76 older adults who were patients of a daycare center diagnosed with aMCI. A sample of 55 highly educated young adults was also examined in the same experiment to qualitatively compare their performance with that of aMCI patients. Participants were assigned a scenario where a hypothetical partner (either a friend or a stranger) was engaged in a task in which the partner could lie to boost their earnings at the expense of the participant. The results showed that aMCI patients, even if they understood that something was going wrong, did not invest in interpretations of potential deception and tended to avoid searching for confirmative information related to the hypothetical lie of their partner compared to highly educated young adults. It seems that aMCI patients become somehow “innocent”, and this is discussed in terms of cognitive impairment and/or socioemotional selectivity.

## 1. Introduction

Mild cognitive impairment (MCI) is considered to be a transitional stage between normal aging and dementia (Petersen 2004. Memory loss is the predominant feature of MCI, but there may also be deficits in perceptual ability, attention, judgment, and speech that are not justified by age. The most common symptoms seen in people with MCI are issues with episodic memory, visuospatial ability, naming, and executive functions (Panza et al. 2005). Patients exhibit mild cognitive disorders that are perceived by their families, although these do not affect their daily performance (Touchon et al. 2006).

Deficits in attention and executive function are common in MCI, especially in multi-domain aMCI, and are the most significant additional symptom (Johns et al. 2012). According to Ready et al. (2003), it is common for aMCI patients to develop increased executive impairment. In particular, tasks requiring executive control are essentially affected in MCI patients and are reliable predictors of Alzheimer’s disease and dementia before diagnosis (Rapp and Reischies 2005). It seems that theory of mind (ToM) abilities are also affected in MCI patients, especially those that require the concurrent processing of linguistic and paralinguistic input (Tsentidou et al. 2021). According to Moreau et al. (2015), aMCI patients have difficulties deducing and attributing others’ behavior in real-life interactions. In comparison with a control group, MCI patients showed deficits in ToM in the early stages of cognitive impairment, which may impact precocious relationships in daily life. 

The early detection of MCI symptoms is essential for the prognosis of Alzheimer’s disease, which is the most common type of dementia (Morris et al. 2001). However, not all patients with MCI develop dementia, although the most known type of MCI, the amnestic type of MCI (amnestic MCI-aMCI), is considered to be a prognostic factor for Alzheimer’s disease and dementia (Fischer et al. 2007). On the other hand, several factors have been identified as contributing to the development of MCI in Alzheimer’s disease, such as the presence of the APOEε4 allele gene, which plays a key role in the overall course of the disease (Petersen et al. 1995). Nevertheless, it has been shown that cognitive reserve, physical activity, and a healthy lifestyle can play a protective role in the progress of MCI (Stern 2012).

According to Winblad et al. (2004), there is great heterogeneity in the manifestation of MCI in clinical practice, which is why four subtypes of it have been identified. Classification is based on the number of cognitive fields affected (single or multiple) and the type (memory, non-memory, or both) of MCI. Thus, the four subtypes of MCI are (1) amnestic MCI, where memory impairment is the only symptom; (2) multiple-domain amnestic MCI, where memory impairment is observed together with impairment in one or more additional domains; (3) non-amnestic multiple-domain MCI, with deficits in more than one non-memory-related domain; and (4) non-amnestic MCI, with deficits in a single non-memory-related domain (Golomb et al. 2004).

The broader diagnosis of MCI is based on the criteria of Petersen (2004), which are subjective memory complaints, preferably confirmed by someone else; greater memory impairment than expected based on age as well as educational level; maintenance of global cognitive and daily function; and absence of dementia.

## 2. Interpretation of Deception

Deception, or the ability to provoke false beliefs in those around us, is often observed in social interaction (El Haj et al. 2017). Deception is defined as the psychological process in which a person attempts to persuade someone else to receive as true what they know to be false in order to gain some benefit or avoid some loss (Abe 2009). The two parameters that constitute the construct of deception are the production of a lie and the detection of it (Zuckerman et al. 1981). At the research level, most studies focus on detecting lies because of their usefulness for clinical, legal, and ethical purposes. Researchers have identified a variety of nonverbal cues related to deception, such as subtle changes in facial expression, tone of voice, and posture (Frank and Ekman 1997; Vrij 1994). It is common knowledge that lies always tend to be selfish, whereas “white lies” tend to be altruistic and social (Abe 2011). In fact, the majority of people are truth-biased in their social contacts. This means that people have a tendency to believe what is said by others and this is thus a default cognitive state. Truth-bias and truth-default states are adaptive and enable fruitful communication. However, truth bias makes people vulnerable to occasional deception (Levine 2014).

Regarding the cognitive dimension of deception, it is considered one of the “higher-level” cognitive or executive functions, which are planning, alerting, switching, suspending, finding, and implementing strategies. The sum of executive functions is the “cognitive control” of behavior (Bryan and Luszcz 2000). Neurophysiologically, activation is limited to specific areas of the prefrontal cortex, which may be related to the suppression of truth and the production of lies (Spence et al. 2001). According to Mohamed et al. (2006), 14 areas of the brain are more active in producing lies than truth. Most of these areas are related to inhibition and attention (anterior cortex), social intelligence (lower parietal lobe and middle frontal helix), or emotion (limbic system). However, activation was also observed in areas that were associated with language processing. On the other hand, it has been shown that patients with problems in the right hemisphere, especially in the middle prefrontal cortex, have a reduced ability to detect lies, and this is associated with deficits in theory of mind (ToM) abilities (Stuss et al. 2001).

According to Abe and Greene (2014), when people participate in a cash prize game, they show increased prefrontal magnetic resonance imaging (fMRI) activity, responding to opportunities presented to them to win by dishonest means. Furthermore, during responses to insincere statements, participants show increased activity of the nucleus accumbens, which associates the limbic system with reasoning processes (Abe and Greene 2014). 

Similarly, the crucial role of the nucleus accumbens in deception has been highlighted by recent research that also focuses on fMRI findings showing that intense activity in the sloping nucleus is associated with the tendency of individuals to deceive others (Speer et al. 2020).

In terms of deception, previous research has demonstrated abnormal trust and deceptive behaviors in children with autism spectrum disorder (ASD) in comparison to neurotypical children. ASD children had no difficulty identifying deception when the social cues were replaced by physical cues, while they could not interpret deceptive behavior through social interaction. These findings support the proposition that the abnormality of trust and deceptive behaviors in children with ASD is mainly due to their defective social skills, as the social cue condition is cognitively more demanding because more information is available for processing (Yang et al. 2017). According to Baron-Cohen (1992), deception impairment in children with ASD indicates how theory of mind (ToM) deficits affect behavior in real social contact.

## 3. Theory of Mind (ToM) and Deception Skills

In addition to executive functions, whose role is crucial to deception ability, theory of mind is of particular importance in both the production and detection of deception (Spence et al. 2001; Sip et al. 2008). The term “theory of mind” refers to the ability to understand and attribute mental states to oneself and others (Premack and Woodruff 1978). It has been shown that ToM consists of two dimensions: cognitive and affective. The cognitive dimension is related to drawing conclusions about the thoughts and beliefs of oneself and others, while the affective dimension is related to drawing conclusions about the feelings of oneself and others (Brothers and Ring 1992; Shamay-Tsoory et al. 2006). The test of misconceptions is a widely used tool for assessing the cognitive dimension of ToM and examines the understanding that one‘s belief may conflict with reality (Bauminger-Zviely 2013). Many researchers argue that the acquisition of ToM is a prerequisite to be able to deceive since in order to instill a false belief in another, one must first have mastered its meaning (Moses and Flavell 1990) but must also be in the position of the deceiver to monitor and control through the transmitter’s reactions if their lie becomes credible (Vrij et al. 2010).

Poletti et al. (2011) approached the relationship between deception and ToM by studying the case of a patient with frontotemporal dementia who pathologically lied. This specific patient showed reduced sensitivity and a lack of concern about deceiving. This can be attributed to reduced ToM skills, which is due to dysfunction of the dorsolateral and medial prefrontal cortex and the orbitofrontal cortex.

## 4. Relationships between Executive Functions and ToM

According to Carlson and Moses (2001), two theories could explain the relationship between executive functions and TοΜ. On the one hand, executive functions may affect the expression of already acquired TοΜ skills as most TοΜ projects require specific cognitive functions, such as inhibition. On the other hand, the development of executive functions may be required before an individual manages to construct complex mental concepts.

Finally, regarding the effect of age on the relationship between executive functions and ΤοΜ, it has been shown that in seniors, the age factor and the speed of information processing affect the aforementioned relationship (Duval et al. 2011).

The study of the ability to deceive in older age is based on the well-established knowledge that executive functions are impaired with age (Hasher and Zacks 1988). Research has revealed significant age differences in both the ability to cheat and the detection of cheating, which may be due to neurobiological changes (Debey et al. 2015). According to Stanley and Blanchard-Fields (2008), older adults over 60 years of age were found to perform worse in detecting lies than younger adults. In addition, they performed worse in producing fraud. In the relationship between age and errors in deception, the reduced ability to recognize emotions that is presented by older people has a mediational role (Slessor et al. 2014).

## 5. The Purpose and Hypotheses of this Study

The aim of the current study was formulated based on a previous experiment examining the motivations for the ascription and interpretation of insincere behavior in young adults, which showed that individuals tend to interpret the behavior of strangers as more insincere than the behavior of friends. Simultaneously, individuals tend to seek information to confirm the insincerity of an act more when it comes from strangers than when it comes from friends (Vainapel et al. 2021).

In this light, the present study focuses on examining the motives for interpreting deception in a specific population, that is, in patients with aMCI, in order to examine the relationships between their cognitive deficits due to neurodegeneration and the interpretation of potentially deceptive interactions. To enhance the study of the aforementioned issue, an additional sample of young, highly educated adults was included in the study to facilitate a qualitative comparison between the two groups: aMCI patients and a group of individuals at the peak of their intelligence in terms of both fluid and crystallized dimensions of it.

The hypotheses of this study were formulated as follows:

**Hypothesis** **1:** *Due to deficits in cognition and social cognition, aMCI patients will not be able to properly detect others’ deceptive intentions. aMCI patients will not be capable of discerning that the higher the reported dice score, the more likely it is that their partner is lying in order to benefit monetarily.*

**Hypothesis** **2:** *aMCI patients will not be more suspicious of a stranger than they are of a friend, they will not deem result statements as likely to be insincere, and they will not tend to search for information that confirms the insincerity of the statements.*

**Hypothesis** **3:** *Young adults with high cognitive potential will appropriately distinguish between when someone may or may not be motivated to lie. Additionally, they will be more likely to perceive the statements of a stranger as insincere compared to those of a friend. Hence, in a qualitative comparison of the two samples, aMCI patients will display lower performance in detecting and processing deception compared to young adults with high cognitive potential.*

## 6. Method

### Participants

The main sample of this study comprised 76 older adults given an aMCI diagnosis (either single- or multi-domain) given by specialized health professionals of “Agia Eleni” Day Care Center in the last six months (40 men and 36 women, age range: 60–87 years, M = 69.71, SD = 5.50). They were randomly selected from the database of the “Agia Eleni” Day Care Center of the Hellenic Society of Alzheimer’s Disease and Related Disorders. For comparison reasons, another sample of 55 young adults with high cognitive potential (21 men and 34 women, age range: 19–27 years, M = 22.29, SD = 1.83) was also examined. At this point, it should be mentioned that the comparison could only be qualitative at this phase because in a quantitative statistical comparison of these groups (healthy, highly educated young adults vs. older adults diagnosed with aMCI, mostly with a lower level of education), age and educational level would act as confounding variables. Regarding the educational level of the young adults, all participants (100%) were highly educated (13 or more years of education). However, concerning the aMCI patients’ educational level, 49 individuals (64.5%) had a medium educational level (7–12 years of education) and 27 individuals (35.5%) were highly educated (13 or more years of education).

Based on a power analysis that was conducted for the aMCI group using G*Power (Faul et al. 2007), a total sample size of 64 participants was recommended to detect an effect size of 0.25 with an alpha of 0.05 and achieve a power of 0.80. The total score for the Montreal Cognitive Assessment Scale (Poptsi et al. 2019; Nasreddine et al. 2005) was used to assess the general cognitive status of aMCI patients (score range: 19–30, M = 25.74, SD = 2.45). Regarding their allocation to the experimental conditions, participants were asked to evaluate a potentially deceptive statement: 19 participants (25%) evaluated statements from a male friend, 18 participants (23.7%) evaluated statements from a female friend, 19 participants (25%) evaluated statements from a male stranger, and 20 participants (26.3%) evaluated statements from a female stranger. The same allocation method was followed for young adults as well: 13 participants (23.63%) evaluated statements from a male friend, 15 participants (27.27%) evaluated statements from a female friend, 14 participants (25.45%) evaluated statements from a male stranger, and 13 participants (23.63%) evaluated statements from a female stranger. The inclusion criteria for aMCI patients were the following: (a) diagnosis of a minor neurocognitive disorder according to the DSM-5 (American Psychiatric Association 2013); (b) the disease corresponds to the third stage of the Global Deterioration Scale, GDS); (c) 1.5-degree standard deviation (SD) below the mean of age and education standards for the field of memory as measured by the neuropsychological tests; and (d) the absence of clinical depression and anxiety as assessed by neuropsychological evaluation. aMCI diagnoses were based on neurological examination, neuropsychological and neuropsychiatric assessments, neuroimaging, and blood tests.

## 7. Measures: The Experiment

The participants, after being informed about the purposes of the study, consented to participate and their demographic data were collected. During the presentation of the experiment scenarios, participants were randomly divided into four conditions that were related to the scenario that would be presented to them: (a) male friend, (b) female friend, (c) male stranger, and (d) female stranger. 

Participants were initially asked to imagine that they were playing a dice game with a teammate, friend, or stranger—male or female—depending on the condition they were randomly assigned. This was followed by three exploratory questions aimed at confirming that the person the participant had in mind met the criteria of the condition they were asked to imagine.

The scenario instructions stated that the person whom the participants were asked to imagine, namely, their alleged teammate, would roll dice, see the result of the roll, and then report it. The result of their roll would determine the payment for both parties. As for the imaginary subject’s payment, the amount they would receive was equivalent to the number they got on the roll. As regards the teammate, namely, the study participant, the amount they would win was derived from the arithmetic formula 6-X (where X is the roll number). Participants were given an example with the number “2” as the result of the dice roll. So, if the roll was 2, then the imaginary subject would win EUR 2.00, while their teammate–study participant would win EUR 4.00 (6–2 = EUR 4.00). 

Once the scenario had been described, participants were asked to answer four comprehension questions to verify that everyone in the study understood the instructions. Participants were then presented with three possible dice rolls (numbers 1, 6, or 3). These numbers were selected as results of the dice roll because according to the game rules, if the outcome was equal to 6, the imaginary subject would win (EUR 6.00 for them/6–6 = EUR 0.00 for the teammate–participant), so they had a reason to lie (to assert that the result was “6”) to gain more money. On the other hand, if the outcome of the dice roll was number 1, the imaginary subject would be at a disadvantage (EUR 1.00 for them/6–1 = EUR 5.00 for the teammate–participant). Number 3 as a possible outcome was an intermediate outcome.

In each of the outcomes mentioned above, the participants were asked (a) to estimate what the partner’s actual roll outcome was, (b) whether they thought the result they reported was a lie (on a Likert scale of 1 = not at all to 7 = very much), and (c) whether the participant would like to see the roll themselves in order to verify whether their teammate’s statement was valid (on a Likert scale of 1 = not at all to 7 = very much). To ensure that the order of presentation of the possible results was not biased (depending on whether the result presented first was in the interest or not of the teammate), half of the participants were firstly presented with the highest roll, the number 6, and for the other half of the participants, the first possible result was the lowest roll, number 1, regardless of the condition in which they were distributed. 

Finally, participants were asked to describe the appearance of the person they had in mind to investigate whether there was bias in the stranger condition regarding whether one would be suspicious of their teammate.

## 8. Procedure

All the aMCI patients had undergone a neurological examination by a neurologist and a neuropsychological assessment by a neuropsychologist. Based on the diagnosis, they were asked to participate voluntarily in the study. Young adults were informed through social media about the purposes of the study and, after giving their consent, took part in the study. The scenario of the experiment was presented online to the participants by a trained psychologist and the duration of the experiment was 15 min.

## 9. Ethics Statement

After all participants had been made aware of the study, they provided their written consent, acknowledging that the research team could use their data for research purposes.

This study was approved by the Scientific and Ethics Committee of Alzheimer Hellas (67-8/17-04-2021), which follows the guidelines of the new General Data Protection Regulation (EU) 2016/679 enacted by the European Parliament and the Council on 27 April 2016. This regulation ensures the protection of individuals’ personal data during processing and promotes the unrestricted movement of such data. Furthermore, the study abided the principles outlined in the Helsinki Declaration.

## 10. Statistical Analysis

The data analysis was conducted via SPSS software version 25 (IBM Corp., 2017, Armonk, NY, USA) and EQS version 6.4 (Bentler 2006). The analyses carried out were (a) multivariate analysis of variance (MANOVA), (b) mixed-measures ANOVA, and (c) path analysis. The main aim of the MANOVA was to examine whether there were potential effects of gender and educational level on the aMCI participants’ performance. The aim of the mixed–measures ANOVA was to examine if the “number” of the dice and the type of the partner affected the estimations of each of the two samples regarding potential deception. At an additional level, path analysis was used for the data of the aMCI group to examine if the exact age in years and the total score for the Montreal Cognitive Assessment (MoCA) affected directly or indirectly the dice score’s reliability estimation and confirmation estimation, respectively. In assessing the fit between a model and the data, several indicators are considered. One such indicator is the χ^2^, where a non-significance level (*p* < 0.05) suggests a good fit with the data (Bentler 2006). Another indicator, the comparative fit index (CFI), compares observed data with a hypothesized measurement model. A CFI value greater than 0.90 indicates an adequate fit of the model with the data, while values approaching 1.00 suggest a very good fit (Hu and Bentler 1999). The standardized root mean square residual (SRMR) index is an additional measure used to assess how well a model fits the observed data. A smaller SRMR value indicates a better fit of the model with the data, with values closer to zero indicating a more accurate fit. An additional indicator is the root mean squared error of approximation (RMSEA). A value below 0.05 indicates a good fit of the model with the data (Bollen 1990). 

## 11. Results

When conducting the first MANOVA concerning the aMCI group, we used the variables “gender” (two levels: male, female) and “educational level” (two levels: middle (7–12 years of schooling), high (13 or more years of schooling)) as the independent variables. We considered the dice score’s reliability estimation (three levels) as the dependent variable. The results of the MANOVA showed that the main effects of gender and education as well as their interaction effects were not significant (*p* > 0.05).

**Figure 1:**
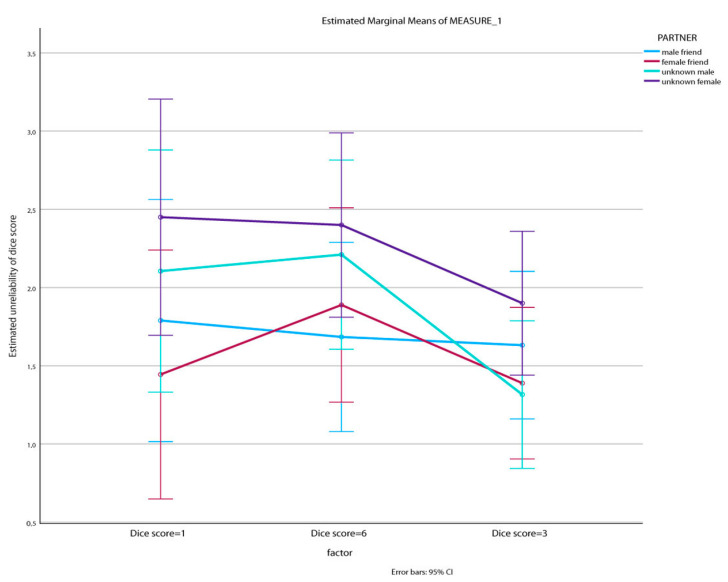
The effects of type of partner and dice score on the evaluation of the unreliability of the dice score for the aMCI sample.

Then, a 3 × 4 mixed-measures ANOVA with dice score reliability estimation (three levels) as the within factor and the experimental condition (four levels) as the between factor revealed a significant main effect of the within factor (F (2, 76) = 8.203, *p* < .001, η^2^ = 0.188). Pairwise comparisons concerning the dice score reliability estimations showed a significant difference between dice score “6” and dice score “3” (I–J = −0.487, *p* = .004), which showed that aMCI participants were more suspicious when a dice score of “6” was reported in comparison with a dice score of “3”. The experimental condition effect was not significant. Even though the interaction effect was also non-significant (*p* > 0.05), as depicted in Figure 1, it seems that there was a general tendency towards distrust for the female stranger. The same tendency regarding the male stranger was obvious when the dice scores were 1 and 6 as well.

Regarding the group of young adults with high cognitive potential, a 3 × 4 mixed-measures ANOVA with dice score reliability estimation (three levels) as the within factor and the experimental condition (four levels) as the between factor revealed a highly significant main effect of the within factor (F (2, 55) = 153.545, *p* < .001, η^2^ = 0.860). Pairwise comparisons concerning the dice score reliability estimations showed a significant difference between dice score “1” and dice score “6” (I–J = −3.880, *p* < .001), which showed that participants were less suspicious when a dice score of “1” was reported in comparison with a dice score of “6”. Furthermore, a significant difference between dice score “6” and dice score “3” was also revealed (I–J = 2.298, *p* < .001), which indicated that participants were more suspicious when a dice score of “6” was reported compared to a dice score of “3”. Finally, a significant difference between dice score “1” and dice score “3” was found (I–J = −1.582, *p* < .001), which showed that participants were less suspicious when a dice score of “1” was reported in comparison with a dice score of “3”.

Concerning the confirmation of the dice roll outcome for the aMCI group, a second ANOVA was applied with “gender” (two levels: male, female) and “educational level” (two levels: middle (7–12 years), high (13 or more years)) as the independent variables. Dice score confirmation estimation (three levels) was the dependent variable. The main effects of gender and educational level were not significant. Regarding the interaction effect (gender x educational level), it was found to be significant (F (3, 76) = 3.970, *p* = .011, η^2^ = 0.145), but, in the subsequent analyses for each of the three levels (F (1, 76) = 1.168, *p* = .283, η^2^ = 0.016; F (1, 76) = 1.106, *p* = .297, η^2^ = 0.015; F (1, 76) = 2.241, *p* = .139, η^2^ = 0.030), no significant results were found. 

After that, a 3 × 4 mixed-measures ANOVA with dice score confirmation estimation (three levels) as the within factor and experimental condition (four levels) as the between-subjects factor revealed no significant main or interaction effects (*p* > .05).

**Figure 2:**
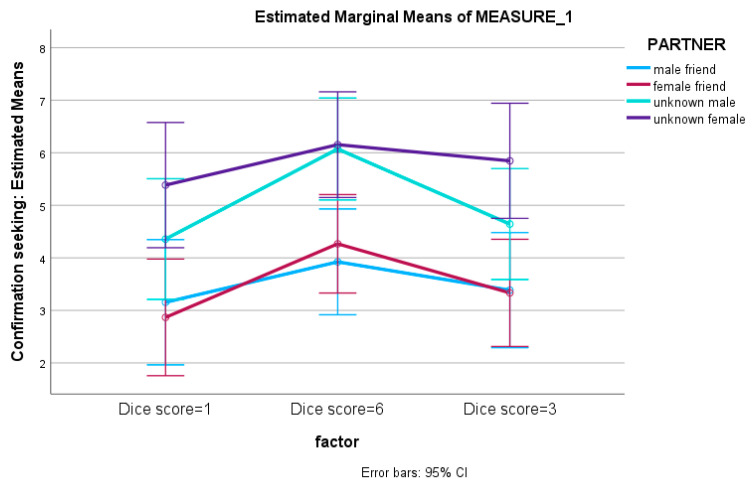
The effects of type of partner and dice score on the confirmation of the unreliability of the dice score for the young adult sample.

With regard to the confirmation of the dice roll for the young adult sample, a 3 × 4 mixed-measures ANOVA with dice score confirmation estimation (three levels) as the within factor and experimental condition (four levels) as the between-subjects factor revealed a significant main effect of the within factor (F (2, 55) = 10.175, *p* < .001, η^2^ = 0.289) as well as a significant main effect of the between factor (F (3, 55) = 6.311, *p* = .001, η^2^ = 0.271). Pairwise comparisons regarding the experimental condition (four levels) revealed a significant difference between female stranger and male friend (I–J = 2.31, *p* = .012). Furthermore, a significant difference was found between female stranger and female friend (I–J = 2.31, *p* = .009). Consequently, it seems that young adults require confirmative information about the reliability of results reported by an unknown woman more than from a friend, whether male or female. For the pairwise comparisons of dice score confirmation estimation (three levels), a significant difference between dice roll “6” and dice roll “1” was found (I–J = 1.163, *p* < .001). This suggests that young adults with high cognitive potential were more likely to seek confirmation of the dice roll result when the result was “6” compared to when it was “1”. In addition, a statistically significant difference was identified between dice roll “6” and dice roll “3” (I–J = 0.802, *p* < .001), which indicates that young adults required confirmatory information to a greater extent for dice roll “6” compared to dice roll “3” (Figure 2).

In the last step, to examine if exact age and total score for the Montreal Cognitive Assessment Scale (MoCA) affected the evaluation of dishonesty by aMCI patients, we ran a path analysis with exact age as the predictor, MoCA total score as the mediating variable, and dice score reliability estimation as the outcome variable. In this case, no path model was confirmed. A similar path analysis was conducted regarding dice score confirmation estimation. Again, no such model was confirmed. These results showed that exact age after 60 years and MoCA score as an index of global cognitive impairment cannot affect, either directly or indirectly, the performance of aMCI patients—neither for evaluating dishonesty nor for confirmation estimations.

## 12. Discussion

The present study focused on examining how aMCI patients differentiate between truthful and deceptive communications and their motives for interpreting deception. The hypotheses of this study were partly confirmed. 

As we hypothesized (Hypothesis 1), potentially due to deficits in cognition and social cognition, aMCI patients had difficulties in properly detecting deceptive intentions. aMCI patients doubted the sincerity of the potential outcome of “6”—where there was a motive for the partner to deceive in order to gain a profit—but they also evaluated dice score “1” as a lie, despite the fact that this result was not beneficial to the potential deceiver. In dice score “3”, which was the neutral condition, they did not demonstrate a greater lack of confidence towards their partner as they estimated that the other person did not have a motivation to deceive them. Potentially, impairment regarding their diagnosis (aMCI), with deficits in theory of mind and executive function (Tsentidou et al. 2017), complicates the process of interpreting others’ intentions. Participants did not properly estimate the potential motives of the deceiver in each condition. Consequently, they estimated dice score “1” to be a possible lie.

On the other hand, concerning the interpretation of such a finding, this finding could be attributed to socioemotional selectivity, that is, as people get older, they tend to focus on positive stimuli and ignore negative ones. They prioritize achieving emotional gratification in the present and do not pay great attention to negativity (Barber et al. 2016). Therefore, a potential explanation for the fact that participants evaluated dice score “1” as a lie is because they were of the opinion that others had positive intentions towards them. 

According to Hypothesis 2, it was expected that aMCI patients would not suspect strangers (males and females) of lying, in contrast with friends. Consequently, they would not search for more information to confirm the insincerity of the strangers’ statements. This hypothesis was partially confirmed as no significant findings were derived. Nonetheless, a tendency of mistrust towards strangers, especially female ones, arose. The type of relationship between the individual and the teammate seemed somehow to affect their motivation to judge behavior as dishonest or not. Due to this, it seems that aMCI patients tended to believe that their friends did not have intentions to deceive them, in contrast to strangers (Vainapel et al. 2021).

Although aMCI patients can detect a deceptive motive, when it comes to processing and ascribing it, they face difficulties, potentially due to ToM deficits. They evaluate other humans’ statements without taking the terms of their relationship and the way it could affect the statement into consideration. This could also be due to the fact that even if they have cognitive schemes about the phenomenon of deception (Shalvi et al. 2012; Vainapel et al. 2021), they cannot easily combine all the information since this requires complex cognitive processes such as executive functions, which are conceptualized as higher-order cognitive capacities (Stuss and Levine 2002). ToM deficits refer to the inability to infer someone’s perspective about reality and elucidate others’ behavior (Moreau et al. 2015). According to Yamaguchi et al. (2012), the performance of aMCI patients in ToM tasks is worse than the performance of healthy controls. Both cognitive and affective ToM are impacted in aMCI patients (Moreau et al. 2015; Poletti and Bonuccelli 2013). Recognizing malicious intentional acts (Yamaguchi et al. 2012) and comprehending phenomena such as sarcasm and metaphor are impaired in aMCI patients (Maki et al. 2013). The decrease in ToM abilities observed in aMCI patients could be responsible for their cognitive deficits, particularly those in memory (Rossetto et al. 2018).

Furthermore, truth bias could significantly affect how aMCI participants perceive and detect deception. Individuals may overlook inconsistencies as they are predisposed to trust what is presented to them, and this can be very well supported by socioemotional selectivity, as mentioned above. People influenced by truth bias may take longer to recognize deception in a situation. They may initially accept false information as true and only later realize that they have been deceived, especially if there is evidence of the specific lie (Bond and DePaulo 2006).

The results of this study also suggest a tendency of aMCI patients to be more suspicious when their partner is a female stranger than when they are a friend or male stranger. Hence, it seems that gender stereotypes may influence those individuals’ perceptions about honesty to some extent. Previous studies have found that humans rate women as more likely to be deceivers than men, especially when the lie is presented as essential or justified (DePaulo et al. 1996). However, people are more likely to suspect women of deceiving than men when the deception is related to emotional or interpersonal issues (Bond and DePaulo 2006) as compared to financial ones. 

Concerning the qualitative comparison of cognitively impaired aMCI people with young adults of high cognitive potential, in terms of their high fluid intelligence due to their age and high crystallized intelligence due to their high educational level (Gongora et al. 2020), Hypothesis 3 was confirmed. Certainly, young adults demonstrated a more precise assessment of the motives for deception compared to the aMCI patients. They demonstrated heightened suspicion of dice roll “6”, where there was a motive for their partner to deceive to gain a profit. Conversely, they exhibited less suspicion of dice roll “1”, where there was no profit incentive for their partner. In the case of dice roll “3”, which represented an intermediate result, they were more suspicious than for dice roll “1”, but not to the extent observed for dice roll “6”. 

Furthermore, it was observed that they predominantly sought confirmatory information in the case of dice roll “6” when there was a potential motive for their partner to deceive them compared to the other dice rolls. Notably, a significant discovery is that the nature of the relationship with the partner played a crucial role in the estimations made by the young adults. Specifically, when the partner was a female stranger, they sought for confirmatory information to a greater extent than they did with friends, whether male or female.

## 13. Conclusions

In conclusion, the present experiment suggests that despite the fact that aMCI patients are more suspicious when there is likely to be a motive for a partner to deceive them, they seem to face specific difficulties in processing the motivations of deception. Conversely, a qualitative comparison with a sample of young, highly educated adults revealed that young adults with high cognitive potential can make accurate estimations regarding the risk of deception.

At the interpretation level, considering both cognitive impairment and socioemotional selectivity aspects, the findings that aMCI patients may become “innocent”—in terms of not suspecting that others would be likely to deceive them—potentially demonstrate the adaptive value of cognitive disruption since it may necessitate selections related to affective wellbeing in the present. The finding that aMCI patients tend to be more suspicious of an unknown female partner, along with the finding that young adults sought confirmatory information about the female stranger’s statements, possibly indicates a gender stereotype linked to the cultural characteristics of Greek traditional society. Past studies in the Greek population have indicated that individuals evaluated exclusively males rather than females as wise people. Indicatively, according to Greek mythology, “Athena”, the goddess of wisdom, was a woman, but was born from Zeus’s head (Moraitou 2002).

## 14. Limitations and Future Research

Despite the gravity of the current results, the limitations of this study should be emphasized. Τhe sample sizes could have been larger. In addition, the MCI participants’ diagnoses were exclusively of the amnestic MCI type. The comparison group used in this study did not permit quantitative comparisons due to confounding variables (sample characteristics). This issue did not afford us the opportunity to elucidate whether the suboptimal performance of the aMCI group could be attributed to cognitive impairment, a lower level of education, or simply the aging process, given the divergence in age and educational levels between the two groups. Consequently, we were unable to discern whether the observed results stemmed from the neurodegeneration associated with aMCI or were merely a reflection of lifespan development and aging. Hence, in the next step of this study, we will proceed with enhancing the older adult sample, incorporating a control group of healthy seniors matched in age and educational level with the aMCI participants, something that is extremely difficult to achieve in this population. Community-dwelling older adults without factors affecting their cognitive status (such as cardiovascular risk factors) do not represent most of the aging population in our country.

Moreover, only one experimental task was administered, and the measures were only for behavioral type. Hence, a groundbreaking future step will be to examine the neural mechanisms and networks that are responsible for performing deception intentions and their changes during lifespan development and neurodegeneration. Future studies will definitely benefit from examining actual deception–detection scenarios or real-life deception scenes. Additionally, other diagnostic groups, such as early AD dementia or behavioral variant frontotemporal dementia patients, could be added to the sample to examine differences between patient groups. Finally, a longitudinal design using multiple tasks or experiments could significantly add to the knowledge of human nature.

## Data Availability

Data available upon duly justified request.
